# Poster Session II - A227 NEO-TERMINAL ILEUM HISTOLOGY PREDICTS LONG-TERM CLINICAL OUTCOMES IN POSTOPERATIVE CROHN’S DISEASE PATIENTS IN ENDOSCOPIC REMISSION

**DOI:** 10.1093/jcag/gwaf042.227

**Published:** 2026-02-13

**Authors:** S Shen, R Chen, P Olivera, K Borowski, C Hernandez-Rocha, W Turpin, K Croitoru, M Silverberg, R Riddell, J Conner, S Lee

**Affiliations:** Sinai Health, Toronto, ON, Canada; Sinai Health, Toronto, ON, Canada; St. Michael’s Hospital, Toronto, ON, Canada; Sinai Health, Toronto, ON, Canada; Sinai Health, Toronto, ON, Canada; Sinai Health, Toronto, ON, Canada; Sinai Health, Toronto, ON, Canada; Sinai Health, Toronto, ON, Canada; Sinai Health, Toronto, ON, Canada; Sinai Health, Toronto, ON, Canada; Sinai Health, Toronto, ON, Canada

## Abstract

**Background:**

Crohn’s disease (CD) is characterized by chronic inflammation of the digestive tract, where surgery may often be required. Postoperative (post-op) recurrence remains common and difficult to predict. Although certain microbial taxa have been identified, other potential predictors, including the histological appearance of the neo-terminal ileal (neo-TI) mucosa, are not well defined.

**Aims:**

This study aims to evaluate the association between histological features from the neo-TI and long-term clinical outcomes in post-op CD patients who are in endoscopic remission.

**Methods:**

In this prospective study, CD patients who underwent ileocolic resection at Mount Sinai Hospital, Toronto, with neo-TI biopsies at ∼6 months post-op were recruited. We included patients in endoscopic remission (mRutgeerts≤i2a) at the 1^st^ post-op colonoscopy. The primary outcome was time-to-clinical recurrence (CR), defined as the presence of CD symptoms and objective evidence of inflammation by imaging, endoscopy, or lab tests (CRP>5 mg/L or Fecal Calprotectin>250 ug/g), within 6 months of onset. Secondary outcomes included time-to-therapy initiation/escalation, CD-related hospitalization, and global recurrence (any of the events above). A comprehensive neo-TI histology assessment items were determined by expert IBD pathologists (J.C., R.R.). Neo-TI histology, including Robarts Histopathology Index (RHI) and various features that are not part of the traditional grading schemes, was evaluated (J.C.), blinded to clinical outcomes. A multivariable Cox proportional hazards model accounting for confounders (e.g., prior resection, post-op biologic use) was utilized.

**Results:**

79 CD patients in endoscopic remission at the 1^st^ post-op colonoscopy was included. Increased RHI was significantly associated with increased CR (adjusted HR = 1.49 per SD, 95%CI: 1.02–2.18, p = 0.041). Biopsy area chronically inflamed was independently associated with increased risk of therapy escalation (aHR=1.74 per SD, 95%CI: 1.21–2.50, p = 0.003). In contrast, increased neutrophils in epithelium (5-50% vs 0% of crypts) was associated with increased risk of CD-related hospitalization (aHR=3.55, 95%CI: 1.05-11.96, p = 0.041). Interestingly, high RHI, determined using an optimized cutoff, demonstrated greater predictive performance for global recurrence (C-index=0.661) compared to the endoscopic activity (mRutgeerts Score) (C-index=0.628).

**Conclusions:**

In post-op CD patients in endoscopic remission, histological features in mucosal biopsies from the neo-TI were associated with long-term clinical outcomes, with higher predictive performance compared to the endoscopic score. Further validation on the role of neo-TI histology as early markers of the post-op disease course is warranted.

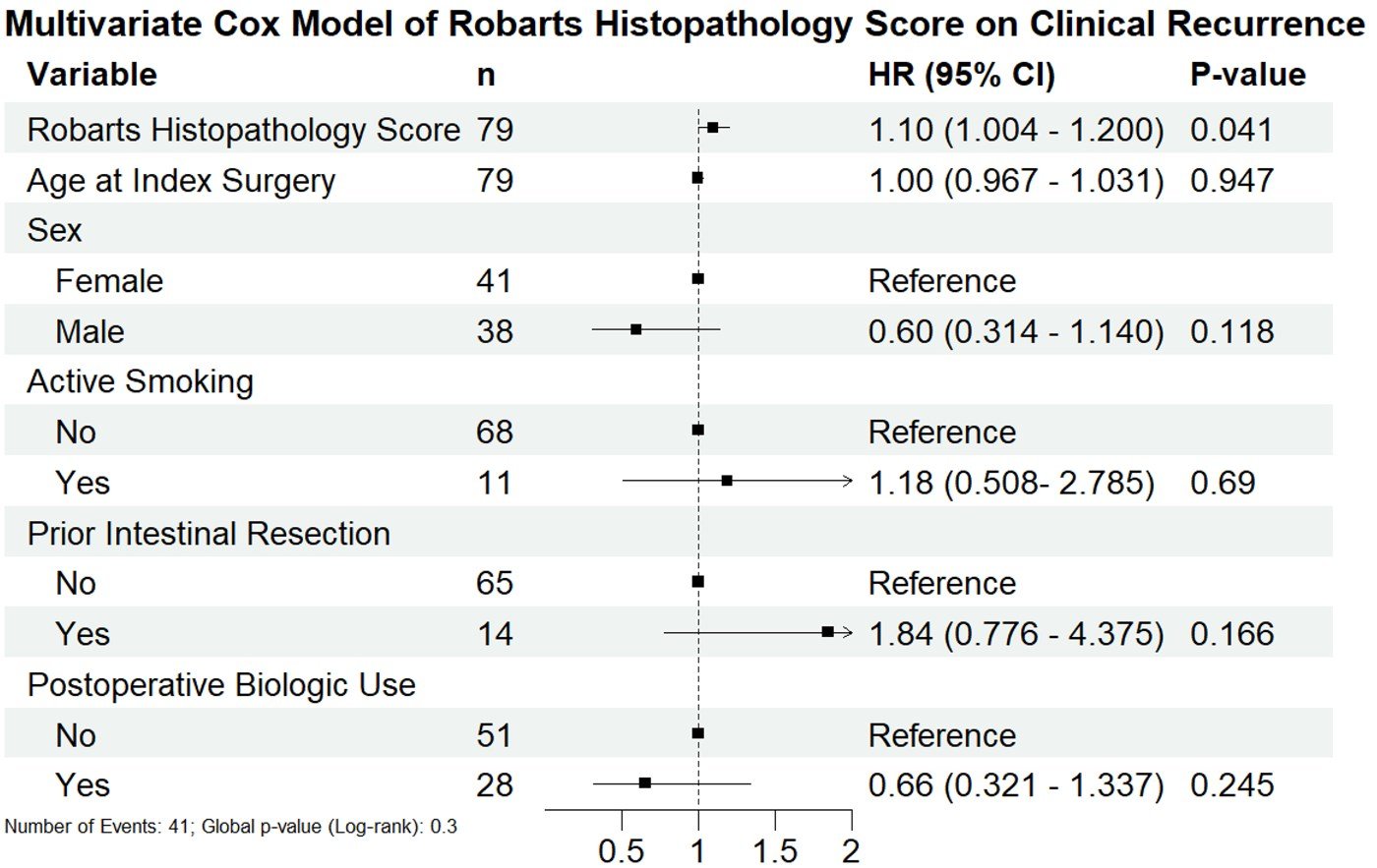

**Funding Agencies:**

CIHRLunenfeld-Tanenbaum Research Institute, NIH NIDDK R01

